# Exposure to bullying behaviours and support from co-workers and supervisors: a three-way interaction and the effect on health and well-being

**DOI:** 10.1007/s00420-019-01503-7

**Published:** 2019-12-11

**Authors:** Stefan Blomberg, Michael Rosander

**Affiliations:** 1 Department of Behavioural Sciences and Learning, Linköping University, 581 83 Linköping, Sweden; 2 Department of Occupational and Environmental Medicine Center, Department of Clinical and Experimental Medicine, Linköping University, Linköping, Sweden

**Keywords:** Workplace bullying, Health outcomes, Social support, Co-workers, Leadership, Moderation

## Abstract

**Purpose:**

Workplace bullying can be very stressful and it has detrimental effects on health and well-being which makes it an important area of study. Social support has traditionally been seen as important in moderating work-related stress. It was hypothesised that the negative association between exposure to bullying behaviours, and health and well-being is moderated by (a) perceived support from close co-workers and (b) perceived supportive leadership. In the study, we also investigated a three-way interaction between exposure to bullying behaviours, perceived support from close co-workers and perceived supportive leadership. This association has not been studied before and add new knowledge to the research field.

**Methods:**

We used a moderated moderation analysis of workplace bullying, co-worker support and supervisor support using cross-sectional data from a work environment survey with 1383 respondents (75% response rate).

**Results:**

The moderated moderation analysis confirmed the moderating effect of perceived co-worker support but not the moderating effect of perceived supervisor support. There was a three-way interaction, but not in the case of the lowest 12.6% of perceived supervisor support scores.

**Conclusions:**

These results indicate that the negative effect of workplace bullying on health and well-being is weaker if victims perceive that they have co-worker support, but this protective effect seems to be conditional on the perceived level of supervisor support. In other words, lack of supportive leadership may block the beneficial effect of perceived co-worker support.

## Introduction

Workplace bullying has detrimental effects on both individuals (Nielsen et al. 2014) and organizations (Hoel et al. 2011), and finding ways of reducing these effects are of utmost importance. There are a lot of studies about resources in the workplace that can enhance employee well-being and performance (see for example, Nielsen et al. 2017a, b). However, according to Einarsen et al. (2016) there have been little research into organizational factors that may prevent workplace bullying from occurring. There are also few studies investigating factors that may protect individuals against the effects of workplace bullying. Some studies have looked at support from co-workers (see for example Rousseau et al. 2014; Warszewska-Makuch et al. 2015; Van den Brande et al. 2016) and supportive leadership (see for example Gardner et al. 2013; Clausen et al. 2019) as protective factors. However, to our knowledge no previous studies have looked at the effects of support from these two sources in combination. As a supervisor not only has opportunities to influence the work environment for an individual co-worker, but also the work environment as a whole (Nielsen 2013) it is possible that the support from co-workers at least in part is dependent on the supervisor. In this study we investigated the possible protective effects of perceived co-worker support and perceived supervisor support on the anticipated negative health consequences of workplace bullying.

Workplace bullying is defined as a systematic exposure to negative behaviours at work over a period of time, in situations where one is unable to fully defend oneself (Einarsen et al. 2011). About ten percent of the working population is exposed to workplace bullying and it is a serious workplace stressor affecting all levels and all sectors (Zapf et al. 2011). Workplace bullying can be viewed as an escalating process whereby the victim’s exposure to negative acts increases and he or she ends up in an inferior position (Einarsen et al. 2011; Leymann 1996). As the definition of bullying highlights, being exposed to bullying behaviours does not automatically mean a person is a victim of bullying. Notelaers and Einarsen (2013) proposed two cut-off scores for determining how to interpret frequency and amount of exposure in relation to workplace bullying. Rosander and Blomberg (2019) suggested levels of bullying where exposure over the lower cut-off together with a lower frequency was framed as the risk of bullying. However, they showed that already when categorized as in risk of bullying the consequences for the experience of work, and one’s health were distinct compared to the not bullied.

Previous research has established that workplace bullying can have detrimental health effects (Einarsen and Nielsen 2015; Nielsen and Einarsen 2012; Nielsen et al. 2014; SBU 2014), and the consequences of exposure range from unemployment (Glambek et al. 2015), an increased risk for disability pensioning (Nielsen et al. 2017a, b; Clausen et al. 2019) to suicidal ideation (Nielsen et al. 2015). This supports predictions derived from the Social pain perspective (MacDonald and Jensen-Campbell 2011), that ostracism and social exclusion—which can be crucial elements of workplace bullying—may have a severe negative impact on health (Knack et al. 2011). The detrimental health effects of workplace bullying have generally been investigated by measuring ill-health rather than overall health or positive health indicators. This means a risk of missing information about the healthy majority of the population (Bowling 2005). There are exceptions, for example, a large English study (Hoel et al. 2004) found that workplace bullying had a negative impact on health and well-being. More recently, studies from Italy and Spain (Arenas et al. 2015) and from Canada (Trépanier et al. 2013) have also found that workplace bullying has negative effects on psychological health and well-being. In these studies, and in the current study there is a focus on more positive aspects of health using indicators of well-being, rather than focusing on morbidity, diseases or weaknesses, that is, a salutogenic perspective (Millar and Hull 1997). No matter if the measure of health is based on ill-health, or health and well-being there is strong support for the connection between exposure to bullying behaviours and health, which is the starting point for this study—the question is if it is reasonable to assume that support is a moderator.

Workplace bullying has been described as a social stressor (Nielsen and Einarsen 2012), and support is often presented as a key mediator or moderator of work-related stress (Cassidy et al. 2014). Usually, a distinction is drawn between received and perceived support, with perceived support typically having a larger impact on outcomes (Taylor 2011). In the Job Demand-Control-Support model (JDCS) support is seen as a moderator of the effect of control on demands (Karasek and Theorell 1990). The link between bullying and support or, in more general terms, the social climate at the workplace has been studied many times over the years and the results have demonstrated that the two concepts are related (see for example Einarsen et al. 1994; Vartia 1996; Skogstad et al. 2007b; Hauge et al. 2007; Baillien et al. 2008; Baillien and De Witte 2009).

Different behaviours or actions can lead to perceptions of support (Foster 2012). It has been described as (a) emotional support (Cohen 2004; Thoits 1982), for example, actions of empathy and trust; (b) instrumental support (Cohen 2004; Schat and Kelloway 2003; Thoits 1982), for example, help and assistance; (c) informal support (Cohen 2004; Schat and Kelloway 2003; Thoits 1982), for example, advice and guidance; and (d) appraisal support (Thoits 1982), for example, provision of information that an individual can use for self-evaluation. Actions that lead to perceptions of support can be provided by the organization itself, but also by supervisors, co-workers, family and friends.

In the workplace, support can come from different sources. Zapf et al. (1996) distinguished between support from co-workers and support from supervisors. They found different outcomes depending on the source of support—more social inclusion followed co-worker support, whereas supervisor support was connected to less verbal attacks and criticism. Nielsen et al. (2019) studied social support as a whole, but also included support separately from supervisors, co-workers and what they described as non-work-related support as moderators for the association between bullying and mental health. The results showed that both supervisor and co-worker support had a protective effect, however, the effect for co-worker support were only there for women.

Looking only at co-worker support, a Polish study (Warszewska-Makuch et al. 2015) reported that the negative association between workplace bullying and mental health was moderated by support from co-workers. The positive effects of co-worker support were also demonstrated by Van den Brande et al. (2016). They suggested that support from co-workers may be a resource that helps victims to find better ways of coping with workplace bullying. Gardner et al. (2013) showed that high co-worker support was negatively associated with exposure to workplace bullying and that there was an interaction between this support and bullying on the psychological strain. Support, in general, seems to have a positive effect on work climate, and studies focussing on co-worker support seem to indicate a positive influence on both the occurrence of bullying and its consequences. Based on this we propose the following hypothesis:

### Hypothesis 1

The negative association between exposure to bullying behaviours, and health and well-being is moderated by perceived support from close co-workers.

The protective effect of a supportive leadership style has been described as a coping strategy in the context of workplace bullying (Van den Brande et al. 2016). Gardner et al. (2013) also found a protective effect of supervisor support against the consequences of bullying. A supportive leadership style can lower the risk for disability pensioning amongst employees exposed to workplace bullying (Clausen et al. 2019). Warszewska-Makuch et al. (2015) showed that the negative relationship between authentic leadership and workplace bullying was moderated by supervisor support. Finally, an American study (Goodboy et al. 2017) used JDCS (Karasek and Theorell 1990) as the framework for a moderated moderation analysis of workplace bullying and found a three-way interaction between demand, control and support, which implies that empowering and supportive supervisors could boost employees’ influence and control over a demanding work situation and thus also lower the risk of workplace bullying. Support from one’s supervisor seems to have a positive effect on workplace bullying and its consequences. Therefore, we propose the following hypothesis:

### Hypothesis 2

The negative association between exposure to bullying behaviours, and health and well-being is moderated by perceived supportive leadership.

There are examples of studies focusing on more than one source of support (e.g. Nielsen et al. 2019), but the effects of these different sources have been investigated separately, not allowing them to interact. However, there are reasons to believe they could affect each other as the formal position of a supervisor give the leader power to influence both the well-being of individual members of the workgroup, as well as, the overall working climate (Nielsen 2013). To the best of our knowledge, the current study is the first to investigate the interaction between perceived support from co-workers and perceived supportive leadership, as well as their effect on the association between workplace bullying, and health and well-being. Previous research has established that the different sources of support can have a buffering effect on bullying and its consequences, but not how they might interact. The different roles of supervisors and co-workers in an organization, and what these differences mean from a formal as well as more informal position is worth investigating. Thus, we pose the following open question:

### Research question

How do the two forms of perceived support, from supervisors and co-workers, interact in understanding the association between bullying behaviours and health and well-being.

## Method

### Study design and sample

The participants in this study were employees at a Swedish governmental institution. We invited 1846 employees to participate in a web-based work environment survey and received responses from 1383 individuals (the response rate was 75%) ranging in age from 21 to 71 years, with a median age of 45 years for both men and women. The mean age of the sample was 45.0 years (SD= 11.1). Women made up 57% of the sample (age range 21–71 years, *M *= 44.6, SD= 11.1). Men made up 43% of the sample (age range: 22–66 years, *M *= 45.4, SD= 11.0).

The data were collected during a three-week period in March 2015 as a work environment survey carried out regularly by the governmental institution employing the participants. We were given an opportunity to collect data as part of an arrangement by which we also analysed the survey data and reported them back in a form that enabled the governmental institution to act on conditions which were having negative effects on the organization and the employees. All employees had the right to refuse their responses to be used for research purposes. A total of 1504 employees completed the work environment survey but only 1383 granted permission for their data to be used for research.

This study is a part of a research project called WHOLE—Work, Health, Organization, Leadership, Experience. The project as a whole has been approved by the Regional Ethical Review Board in Linköping, Sweden.

### Measures

Data were collected using a comprehensive questionnaire (the Psychosocial work environment questionnaire, PSYWEQ; Rosander and Blomberg 2018). The data analyses in this study used only a small subset of the scales included in the questionnaire.

Exposure to bullying behaviours was measured using the Negative acts questionnaire-revised (NAQ-R; Einarsen et al. 2009), which consists of 22 items describing various negative behaviours that can be perceived as bullying if they occur repeatedly. The Swedish version of NAQ-R was used (Rosander and Blomberg 2018). Responses are given by indicating the frequency with which one has experienced the behaviour described in the item during the last 6 months using a scale ranging from 1 (never) to 5 (daily). In our sample, the internal consistency of NAQ-R as measured with Cronbach’s alpha was 0.85. The possible range of NAQ-R is from 22 to 110 points. In our sample, the range was from 22 to 66. According to the definition of workplace bullying, as well as, suggestions of cut-off scores (Notelaers and Einarsen 2013) and levels of bullying (Rosander and Blomberg 2019), this means that many were exposed to bullying behaviours, but not all were bullied. When referring to workplace bullying of just “bullying” onwards we mean an exposure to bullying behaviours without categorizing any individual result as being workplace bullying or not.

Health and wellbeing were measured with the Salutogenic health indicator scale (SHIS; Bringsén et al. 2009), a Swedish scale that measures 12 different aspects of health, energy and feelings of well-being. Responses are given using six-step semantic differential scales, where 1 indicates complete agreement with the negative description of an aspect and 6 indicates complete agreement with the positive description. The internal consistency of SHIS as measured with Cronbach’s alpha was 0.95.

The other scales used in this study were the Perceived support from close co-workers (PSC) and the Perceived supportive leadership (PSL) from PSYWEQ (see Rosander and Blomberg 2018, for factor analyses and further description of these scales). All measures were selected based on previous validation in a Swedish context.

The PSC scale measures different aspects of support in the social working environment and consists of five statements about one’s interactions and experiences with one’s closest co-workers. It mainly focuses on aspects such as trust, support and sense of security. The internal consistency of PSC as measured with Cronbach’s alpha was 0.89.

The PSL scale measures a supportive leadership style which includes trust and confidence in a leader and consists of ten statements about interactions and experiences with one’s immediate supervisor, mainly focusing on social factors such as trust, getting help or support and sense of security. The internal consistency of PSL as measured with Cronbach’s alpha was 0.97.

Responses to both PSC and PSL are given using a seven-point Likert scale, ranging from 1 (do not agree at all) to 7 (agree completely).

**Fig. 1:**
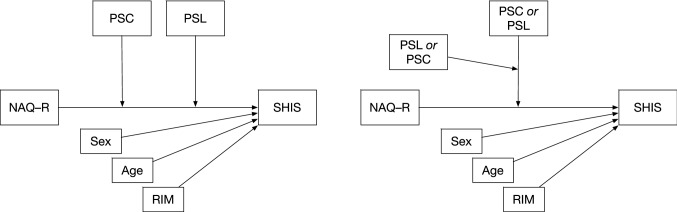
Two conceptual models of moderation including both perceived support from close co-workers (PSC) and perceived supportive leadership (PSL) on the association between exposure to bullying behaviours (NAQ-R), and health and well-being (SHIS). Covariates in the model are sex, age and roles in the organization (RIM)

Sex and age were used as covariates in the analyses. A measure of conflicting and ambiguous roles in the organization (Roles in the organization-RIM; Rosander and Blomberg 2018) based on six items was also included as a covariate. The reason for the inclusion of this covariate is that role problems are recognized as a predictor of workplace bullying (see for example Hauge et al. 2007). The internal consistency of RIM as measured by Cronbach’s alpha was 0.91. In Fig. 1 we present two conceptual models of the relationships between exposure to bullying behaviours, health, perceived support from close co-workers and perceived supportive leadership.

### Statistical analysis

The focus of the data collection was the individual experiences of bullying, health and well-being, and perceived support from co-workers and/or the supervisor. Thus, all data were analysed on an individual level, not on a group or an organisational level. The analyses were carried out using IBM SPSS 24 for Mac together with Hayes’ PROCESS Macro version 3 for SPSS (Hayes 2018). Only 1353 of the 1383 completed surveys had no missing data. No replacement method was used, so the 1353 complete sets of data were analysed in the full model, a 73% response rate based on the original sample.

In the analyses, we tested five different models of increasing complexity: In the first model the expected negative association between exposure to bullying behaviours, and health and well-being was tested. In Model 2 perceived co-worker support was added as a moderator. In Model 3 we tested if the negative association in Model 1 was dependent on perceived supportive leadership. In the fourth model we tested the two moderators in Model 2 and 3 simultaneously, but not letting them interact, and in Model 5 we let them interact with each other. Model 5 was the full model of analysis. As covariates, age and gender were used in all models, and unclear and conflicting roles was used in Model 2 to 5.

In Model 5 a moderated moderation model was tested using the PROCESS macro (Hayes 2018) based on ordinary least squares (OLS) regression analysis of conditional effects (using model 3 in the PROCESS macro). Bootstrapping with 10,000 samples was used to calculate bias-corrected confidence intervals for all the included variables. The dependent variable and the moderating variables were mean centred prior to analysis.

## Results

The means, standard deviations and pairwise correlations for all variables are reported in Table 1. The Perceived supportive leadership (PSL) was positively associated with the Perceived support from close co-workers (PSC) (*r *= 0.41; *p *< 0.01) and the Salutogenic health indicator scale (SHIS) (*r *= 0.31; *p *< 0.01), as well as being negatively associated with the Negative acts questionnaire-revised (NAQ-R) (*r *= − 0.45; *p *< 0.01). PSC was positively associated with SHIS (*r *= 0.27; *p *< 0.01) and negatively associated with NAQ-R (*r *= − 0.38; *p *< 0.01). NAQ-R was negatively associated with SHIS (*r *= − 0.36; *p *< 0.01). Age and sex were not associated or marginally associated with the other variables, but the Roles in the organisation (RIM) was positively associated with PSL, PSC and SHIS, and negatively associated with NAQ-R.

**Table 1:** Descriptive statistics and Pearson’s product-moment correlations for the study variables (Cronbach’s alpha is given in bold along the diagonal)

	*N*	*M*	SD	1	2	3	4	5	6	7
1. Sex	1383	1.43	0.50							
2. Age	1383	44.98	11.06	0.04						
3. NAQ-R	1383	1.17	0.23	0.04	− 0.08*	**0.85**				
4. SHIS	1366	3.84	1.21	0.07*	0.19**	− 0.36**	**0.95**			
5. PSC	1379	6.04	1.01	− 0.01	− 0.02	− 0.38**	0.27**	**0.89**		
6. PSL	1371	5.56	1.41	0.00	0.00	− 0.45**	0.31**	0.41**	**0.97**	
7. RIM	1381	4.90	1.38	− 0.06*	0.10**	− 0.42**	0.34**	0.36**	0.55**	**0.91**
Valid *N*	1353									

Negative acts questionnaire-revised (NAQ-R), Salutogenic health indicator scale (SHIS), Perceived support from close co-workers (PSC), Perceived supportive leadership (PSL), Roles in the organization (RIM)

*p < 0.05, **p < 0.01

### Regression and moderation analyses

A regression analysis was conducted for the association between exposure to bullying behaviours and health and well-being using sex and age as covariates (Model 1). There was a significant negative association between bullying and health (see Table 2). Model 1 explained 16.4% of the variance in health (*F*[2, 1362] = 25.38, *p* < 0.001). Turning to moderation analysis, we followed the procedures described by Hayes (2018) using his first three models. In Model 2 perceived support from close co-workers was added as a moderator of the association between bullying and health resulted in 22.4% explained variance (*F*[6, 1356] = 65.29, *p* < 0.001) and a significant interaction effect (see Table 2). There was also a significant simple effect of co-worker support on health (see Table 2). In Model 3 we tested perceived supportive leadership as a moderator of the association between bullying and health. Model 3 explained 21.9% of the variance in health (*F*[6, 1346] = 63.07, *p* < 0.001). There was a significant interaction effect, as well as a significant simple effect of supportive leadership on health (see Table 2). In Model 4 we tested the two moderators, supportive leadership and support from co-workers, simultaneously, but did not let them interact and thus using them as control for each other. Model 4 explained 23.2% of the variance in health (*F*[8, 1344] = 50.88, *p* < 0.001). The negative effect of bullying on health was significantly dependent on support from co-workers (see Table 2), but not on supportive leadership. There were significant simple effects on health from support from co-workers and for supportive leadership (see Table 2). Finally, a moderated moderation analysis was carried out, letting the two moderators interact with each other. Thereby all three hypotheses and the research question could be analysed simultaneously. The full model explained 24.2% of the variance in health (*F*[10, 1342] = 45.95, *p* < 0.001). There was a significant simple negative effect of bullying on health (*b *= − 1.30, 95% CI [− 1.64, − 0.96], *p *< 0.001). The negative effect of bullying on health was a function of support from co-workers (*b *= − 0.43, 95% CI [− 0.70, − 0.15], *p *= 0.002), which provided support for Hypothesis 1. The negative effect of bullying on health was not, however, a function of supportive leadership (*b *= − 0.07, 95% CI [− 0.23, 0.08], ns), so Hypothesis 2 was not supported. There was a three-way interaction between bullying, support from co-workers and supportive leadership, meaning that the moderation of the negative effect of bullying on health by support from co-workers was a function of the level of supportive leadership (*b *= − 0.13, 95% CI [− 0.22, − 0.47], *p *= 0.002). The significant three-way interaction is discussed below. All five models are presented in Table 2.

**Table 2:** Five regression models of increasing complexity. Five regression models of increasing complexity. Model 5 is a moderated moderation with the Salutogenic health indicator scale (SHIS) as the dependent variable, the Negative acts questionnaire–revised (NAQ-R) as the independent variable and the Perceived support from close co-workers (PSC) and the Perceived supportive leadership (PSL) as moderators. Sex, age and the Roles in the organisation (RIM) are used as covariates

	*b*	95% BCa CI	SE B	*p*
*Model 1 (R*^*2*^* = 0.16)*				
NAQ-R	− 1.87	[− 2.14; − 1.60]	0.13	< 0.001
Sex	0.19	[0.07; 0.30]	0.08	0.002
Age	0.02	[0.01; 0.02]	0.00	< 0.001
*Model 2 (R*^*2*^* = 0.22)*				
NAQ-R	− 1.45	[− 1.76; − 1.15]	0.16	< 0.001
PSC	0.19	[0.12; 0.26]	0.03	< 0.001
NAQ-R*PSC^a^	− 0.33	[− 0.49; − 0.17]	0.08	< 0.001
Sex	0.20	[0.08; 0.31]	0.06	< 0.001
Age	0.02	[0.01; 0.02]	0.00	< 0.001
RIM	0.16	[0.11; 0.20]	0.02	< 0.001
*Model 3 (R*^*2*^* = 0.22)*				
NAQ-R	− 1.44	[− 1.77; − 1.11]	0.17	< 0.001
PSL	0.12	[0.07; 0.17]	0.03	< 0.001
NAQ-R*PSL^b^	− 0.16	[− 0.29; − 0.04]	0.07	0.012
Sex	0.20	[0.08; 0.31]	0.06	< 0.001
Age	0.02	[0.01; 0.02]	0.00	< 0.001
RIM	0.14	[0.09; 0.19]	0.03	< 0.001
*Model 4 (R*^*2*^* = 0.23)*				
NAQ-R	− 1.38	[− 1.71; − 1.04]	0.17	< 0.001
PSC	0.16	[0.09; 0.23]	0.04	< 0.001
PSL	0.08	[0.03; 0.14]	0.03	0.002
NAQ-R*PSC^c^	− 0.30	[− 0.49; − 0.11]	0.10	0.002
NAQ-R*PSL^d^	− 0.04	[− 0.19; 0.11]	0.08	0.623
Sex	0.19	[0.08; 0.30]	0.06	0.001
Age	0.02	[0.01; 0.02]	0.00	< 0.001
RIM	0.13	[0.07; 0.18]	0.03	< 0.001
*Model 5 (R*^*2*^* = 0.24)*				
NAQ-R	− 1.30	[− 1.64; − 0.96]	0.17	< 0.001
PSC	0.20	[0.12; 0.27]	0.20	< 0.001
PSL	0.09	[0.03; 0.14]	0.03	0.001
NAQ-R*PSC	− 0.43	[− 0.70; − 0.15]	0.14	0.002
NAQ-R*PSL	− 0.07	[− 0.23; 0.08]	0.08	0.354
PSC*PSL	0.08	[0.04; 0.12]	0.02	< 0.001
NAQ-R*PSC*PSL^e^	− 0.13	[− 0.22; − 0.47]	0.04	0.002
Sex	0.19	[0.07; 0.30]	0.06	0.002
Age	0.02	[0.01; 0.02]	0.00	< 0.001
RIM	0.12	[0.07; 0.17]	0.03	< 0.001

Note 1: In Model 2 through 5, the dependent variable and the moderating variables were mean centred prior to analysis

^a^The interaction results in a 0.009 increase in *R*^2^, *F* (1, 1356)= 15.87, *p *< 0.001

^b^The interaction results in a 0.004 increase in *R*^2^, *F* (1, 1346)= 6.35, *p *= 0.012

^c^The interaction results in a 0.005 increase in *R*^2^, *F* (1, 1344)= 9.51, *p *= 0.002

^d^The interaction results in no *R*^2^ increase

^e^The interaction results in a 0.005 increase in *R*^2^, *F* (1, 1342)= 9.30, *p *= 0.002

The interaction between support from co-workers and supportive leadership moderated the negative relationship between bullying and health over part of the range of scores for supportive leadership. For the scores for supportive leadership on or above − 1.63 (87.4% of the cases analysed) it moderated the moderation of the relationship between bullying and health by support from co-workers. In Table 3 the conditional interaction between bullying and support from co-workers is presented at three scores for supportive leadership (16th, 50th and 84th percentiles).

**Table 3:** Test of conditional effect of the Negative acts questionnaire–revised (NAQ-R) *the Perceived support from close co-workers (PSC) at 16th, 50th, and 84th percentiles of the Perceived supportive leadership (PSL)

PSL	Effect	*F* (1, 1342)	*p*
− 1.44	− 0.24	4.63	0.032
0.44	− 0.49	10.09	0.002
1.34	− 0.60	10.96	0.001

**Fig. 2:**
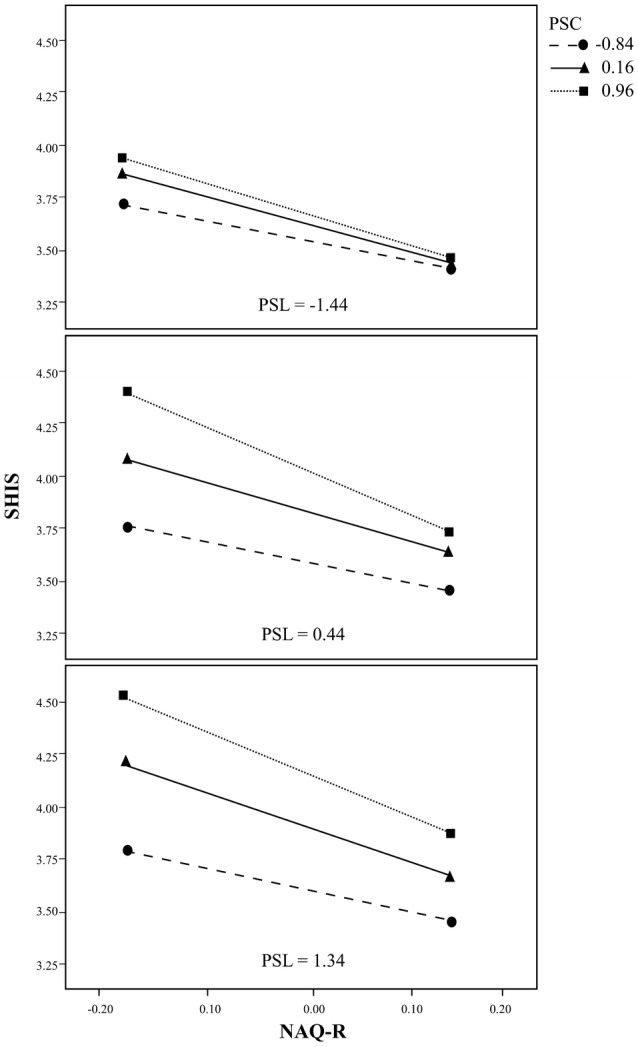
The conditional negative effect of the Negative acts questionnaire-revised (NAQ-R) on the Salutogenic health indicator scale (SHIS) as a function of the Perceived support from close co-workers (PSC) and the Perceived supportive leadership (PSL). The three panels for PSL correspond to values of PSL equal to 16th, 50th and 84th percentiles

The conditional negative effect of bullying on health as a function of support from co-workers and supportive leadership is also presented graphically in Fig. 2 with three panels corresponding to values on supportive leadership equal to 16th, 50th and 84th percentiles.

## Discussion

In this study, we chose to differentiate between perceived support from two different sources within the workplace, that is, close co-workers and immediate supervisors. We were not investigating social support or social climate in general, but the potential effects of perceived support from two clearly differentiated sources. There may still be other sources of support in the organization for example HR personnel or supportive systems in the organization, such as guidelines and procedures that an individual can consult, but they were not included in this study.

The finding that exposure to bullying behaviours is negatively associated with health and well-being replicates what many other studies have showed (for example Einarsen and Nielsen 2015; Theorell et al. 2015; Hoel et al. 2004; Trépanier et al. 2013), but what is more interesting is the interaction effects found.

Perceived support from close co-workers moderated the negative association between exposure to bullying behaviours, and health and well-being. This interaction was negative indicating that perceived support from close co-workers reduced, but did not eliminate, the negative effects of bullying behaviours for the overall health and well-being. The negative interaction also implies that the reduction in negative consequences for health and well-being are likely to eventually disappear at higher levels of bullying behaviours. It is, however, clear that one is less likely to suffer negative effects on health and well-being as a result of workplace bullying if exposure to bullying behaviours arises when one perceives that one has a high level of support from close co-workers.

It is interesting to compare our result with the study by Rousseau et al. (2014). They also found a moderating effect of support from co-workers when investigating how trust in management (as an organizational resource) and role overload (as an organizational demand) affected workplace bullying. They showed that the effects on workplace bullying from both were dependent on factors such as autonomy, employee participation, and support from co-workers. In their study, trust in management had a lowering simple effect on workplace bullying. In cases of low trust, support from co-workers had a buffering (moderating) effect. Even if we did not use the same design, and did not investigate the exact same factors, there are probably important common findings here. They investigated trust in management whereas we investigated perceived supportive leadership, which include dimensions of trust and feelings of security in relation to one’s immediate supervisor. The two factors are similar, however, Rousseau’s et al. (2014) concept is on a general organisational level (general trust) whereas our concept is on an individual level. Another difference is that they investigate the effects on workplace bullying, whereas we investigate the potential moderating effect on the negative association between workplace bullying, and health and well-being. But even if there are clear difference, what is common is the findings that different sources of support and trust in the organisation interact. So, when investigating this phenomenon, one must be careful as the different sources interact and influence each other in important ways.

Surprisingly, in our full model (Model 5) there was no interaction between perceived supportive leadership and exposure to bullying behaviours with respect to health and well-being. This result is the opposite of what one might expect, based on studies which have reported that supportive leadership or trust in management lowers the risks for workplace bullying (see for example Gardner et al. 2013; Rousseau et al. 2014; Van den Brande et al. 2016). However, those studies indicated that supportive leadership reduces the incidence of bullying, whereas our study focused on the effects of workplace bullying on health and well-being. This is more similar to a study by Clausen et al. (2019) who showed a buffering effect of supportive leadership on the risk of workplace bullying leading to disability pensioning. In the current study the Perceived supportive leadership (PSL) and the Roles in the organization (RIM) were highly correlated (*r* = 0.55) and as we used RIM as a covariate we controlled for the effect of conflicting and ambiguous roles in the organization. The lack of a protective effect of supportive leadership in our analysis was at least in part due to the inclusion of RIM as a covariate. This implies that the effect of perceived supportive leadership is linked to how clear the roles in an organization are. In other words, the presence of a perceived supportive leadership may have indirect protective effects mediated by its impact on the organization, rather than having a direct effect at an individual level.

It is also interesting that when the Perceived supportive leadership (PSL) was tested as a single moderator like Clausen et al. (2019) (in Model 3) it actually had a significant moderating effect, but when the Perceived support from close co-workers (PSC) was included (in the Model 4 and 5) the significant moderating effect of PSL disappeared. That, once again, clearly underscores the importance of not investigating support in general and that support from different sources can have different effects, and that there may be significant interactions between these sources of support as we see in our three-way interaction model.

A possible explanation for our finding that there was an interaction between perceived support from close co-workers and exposure to bullying behaviours with respect to health, but not for perceived supportive leadership, can be found in social exchange theory (see for example Coyle-Shapiro and Shore 2007; Cropanzano and Mitchell 2005). Parzefall and Salin (2010) noted that there is a growing body of evidence suggesting that co-workers have an important influence on employees’ perceptions of social relationships. Korte (2009) pointed to the importance of frequency and Wanous (1992) to the quality of social interactions. Svensson (2010) also highlighted the importance of the distance between interaction partners and the regularity of their interactions. One would expect people to interact more frequently with close co-workers than with their immediate supervisor, hence one might expect perceived social support from close co-workers—which may be categorized as a high-quality and high-frequency interaction—to have a stronger protective effect on health and well-being.

Svensson (2010) also showed that proximity and regularity of interaction are important preconditions for bullying behaviours, suggesting that it is important for victims to get out of the way and find a safe place to which they can retreat. One could argue that being exposed to bullying behaviours by people that are physically or socially close, that is, people that one has to interact with on a regular basis, is more damaging than being bullied by people one is more distant to. This implies that if one is bullied by one or more close co-workers it may be very difficult to get the social support that would otherwise have reduced the associated health risks.

Whilst we did not find an interaction between exposure to bullying behaviours and perceived supportive leadership with respect to health and well-being, we did find a three-way interaction between exposure to bullying behaviours, perceived support from close co-workers and perceived supportive leadership. The interesting point is that the moderation of the negative association between exposure to bullying behaviours and health by perceived support from close co-workers is conditional on perceived supportive leadership. By distinguishing between perceived support from co-workers and perceived supportive leadership (see Zapf et al. 1996) we have been able to show that the effects of one are contingent on the other. We also found that when perceived supportive leadership is low the interaction effect disappears. In other words, the health risks associated with being exposed to bullying behaviours in the workplace are reduced if one perceives a moderate or a highly supportive leadership together with support from close co-workers. Conversely, on the lowest 12.6% of the range of perceived supportive leadership, there is no interaction. So, our moderated moderation analysis indicated that the effect of perceived support from close co-workers depends on the level of perceived supportive leadership and, in addition, when the level of the perceived supportive leadership is low perceived support from close co-workers does not moderate the health risks associated with exposure to workplace bullying. Perceiving support from close co-workers will only reduce the health risks associated with exposure to bullying behaviours if one trusts one’s supervisor and feels safe in that relationship.

The finding that lack of trust or security in one’s relationship with one’s supervisor may block the beneficial effects of perceived co-worker support may reflect situation in which the supervisor not only fails to provide supportive leadership but actually acts as a bully (Zapf et al. 2011) which is also discussed by Clausen et al. (2019). It is reasonable to believe that scores in the bottom 12.6% of the range on a scale measuring perceived supportive leadership will include cases in which the respondent is bullied by his or her workplace supervisor. But supervisors are not responsible for all workplace bullying (Zapf et al. 2011). A low score for supportive leadership may reflect circumstances and factors other than a bullying leader. For example, passive and absent leadership, also called laissez-faire leadership, has been shown to be strongly associated with workplace bullying through its effects on role ambiguity, interpersonal conflicts, etc. (Skogstad et al. 2007a).

This highlights the question of who the bully is if one is being exposed to bullying behaviours yet receiving support from both close co-workers and one’s supervisor. In most workplaces, however, there are many organizational levels and many workgroups or social groups, so it is entirely possible that one could be receiving support from close co-workers and one’s immediate supervisor, yet still being exposed to bullying behaviours in the organization (Zapf et al. 2011).

This also opens for questions about the direct and moderated effects on health and well-being of workplace bullying from different sources. It is reasonable that the negative effect of exposure to bullying behaviours may be different depending on if the source is some or all of your co-workers, your supervisor, a client, or a combination of several sources. For example, Törek et al. (2016) showed that employees exposed to workplace bullying from leaders experienced more severe depressive symptoms compared to those that were bullied by co-workers or clients. This result somehow contradicts the reasoning connected to social exchange theory (above) where, for example, proximity (Svensson 2010) and the quality of relationships (Wanous 1992) were of particular importance. One may, however, reason that being bullied is one thing and being protected from its negative effects is another. If perceived support from co-workers is a buffer and protection for the effects on health and well-being from exposure to bullying behaviours, our data suggest that this buffering loses its effect when the perceived supportive leadership is very low. This may suggest that if you are bullied by your supervisor, or that your supervisor knows that you are bullied but doesn’t care, it really doesn’t matter how much of support you get from your co-workers, your health and well-being will suffer anyhow.

It would be interesting to investigate a combination of different settings. For example, bullying from co-workers moderated by perceived support from a supervisor, bullying from some co-workers moderated by perceived support from other co-workers, bullying from a supervisor moderated by perceived support from co-workers etc. Such a refined analysis would, however, demand a longitudinal setting, a very large data sample and very specific questions about who the bullies are. Perhaps it would be better to perform deep interviews and qualitative analysis to further investigate and clarify different aspects of these questions.

Nevertheless, our results call for more research into the questions of how different sources of perceived support and different kinds of support (emotional, instrumental, informal, appraisal; see Foster 2012) may moderate the negative effects on health and well-being of workplace bullying from different sources. However, our study suggests that there may be important differences between perceived supportive leadership and perceived co-worker support, and their protective effects on health and well-being when one is exposed to bullying behaviours.

### Limitations

This study was based on data from a self-report questionnaire. Relying on a single data collection method may lead to common method bias and threaten construct validity (Donaldson and Grant-Vallone 2002) even if such a problem seems to be rarer than has been assumed (see for example Spector 2006). The tendency of an employee to bias his or her response may be evaluated based on four factors (a) the true state of affairs, (b) the sensitivity of constructs, (c) dispositional characteristics, and (d) situational characteristics (Donaldson and Grant-Vallone 2002). On this basis, we conclude that there is little risk that our participants’ responses were biased. For example, participants were not asked to self-report any socially undesirable behaviours among themselves and the constructs were not in itself sensitive for the respondents as we did not use self-definition of being exposed to bullying but the Negative acts questionnaire-revised (NAQ-R)—see Nielsen et al. (2011) for a discussion of the advantages and disadvantages of different workplace bullying measurement methods. Furthermore, the data were collected in the context of a regular work environment survey to which the participants were used to submitting information. This also indicates that data were collected in a context where there was little situational pressure to give socially desirable answers. Also, testing a common latent variable showed only 1.7% common variance among the variables in the study (Podsakoff et al. 2003).

Another limitation is that all our data are cross-sectional which means the directions of the associations between the investigated factors are unknown. Our results were, however, consistent with the theoretical reasoning and other studies behind our hypotheses. Nevertheless, it is possible that, for example, being in poor health could lead to an employee being exposed to behaviours and acts such as being assigned uninteresting and uninspiring work tasks or redeployed because his or her performance has deteriorated and that he or she perceives these events as amounting to workplace bullying. Longitudinal research in which data on bullying and health have been collected on several occasions has concluded that the direction of influence is mainly from workplace bullying to health (see for example Einarsen and Nielsen 2015) although there are circumstances under which the associations may be bi-directional (Einarsen and Nielsen 2015). For example, a chain of events may start with workplace bullying having a negative influence on health, after which the influence flows in both directions because worsening health increases exposure to negative workplace behaviours. We nevertheless conclude from this study that it is more plausible that the negative influence flows mainly from bullying to health, rather than vice versa.

It has been argued that in a moderation analysis the candidate moderators and the predictor variable should be uncorrelated (see Hayes 2018). Clearly, there may be problems with multicollinearity and a high variance inflation factor when these correlations are high. Hayes (2018) argued that it is always a good idea to do what you can to reduce the correlation between the predictor variable and the moderators, but also stated that the non-correlation criterion should be treated as an ideal rather than a requirement. In our data, the correlations between the predictor and the moderators were between 0.38 and 0.45 which might be regarded as less than ideal, but cannot be regarded as high, given that it leaves about 80–85% of variance unaccounted for.

There is only small, although significant increase in R^2^ when adding the interactions when all variables are included in the model. This could of course be due to a rather large sample size. In the model, there are many variables that all, but the interaction between negative acts and perceived supportive leadership, significantly contribute to the explained variance. The final model is the model that explains most variance of the tested models.

A final limitation on our findings relates to their representativeness because our data are cross-sectional and were collected from a single cohort of workers in a governmental department in Sweden. Our results need to be replicated in other employment sectors and other countries.

## Conclusion and practical implications

Exposure to bullying behaviours at work has a negative effect on victims’ health and well-being, but this is reduced if victims receive support from close co-workers. The stronger the perceived support the less severe the negative health effects of exposure to bullying behaviours will be, although support cannot eliminate them altogether. If, however, the target experiences an unsupportive leadership and has little confidence in his or her supervisor then no amount of support from close co-workers will reduce the negative health consequence of the bullying behaviours. The combination of perceived support from close co-workers and perception of supportive leadership produces the greatest reduction in the negative health consequences of exposure to bullying behaviours but still does not provide complete protection.

Our result points to the importance of providing support for individuals exposed to bullying behaviours. An organisation should always act on health risks and making clear that bullying behaviours never can be tolerated. But sadly enough, in many organisations bullying behaviours can be implicitly or explicitly tolerated. Exposed persons should perhaps evaluate their situation and decide whether to stay or seek employment elsewhere. That is, however, not a topic for this study.

As the health risks associated with being exposed to bullying behaviours appear to be considerable, early evaluation of the situation would be valuable. This evaluation could consist of asking oneself two questions (a) do I feel supported by my close co-workers, and (b) do I feel that my supervisor provides supportive leadership? It appears that one is unlikely to cope well with the bullying behaviours unless the response to both questions is positive. Whilst perceived support from close co-workers seems to be more important, it may not have any effect unless one also trusts one’s supervisor.
